# The Role of Cardiac Magnetic Resonance to Predict Response to Cardiac Resynchronization Therapy: A Systematic Review and Meta-analysis

**DOI:** 10.19102/icrm.2024.15111

**Published:** 2024-11-15

**Authors:** Nazima Khatun, Alejandro Sanchez-Nadales, Jonathan Francois, Mohammed Hussein, Muhammed Atere, Yasser Rodriguez, Jose Baez-Escudero, Adam Budzikowski

**Affiliations:** 1 Department of Internal Medicine, SUNY Downstate Medical Center, Brooklyn, NY, USA; 2 Robert and Suzanne Tomsich Department of Cardiology, Department of Cardiovascular Disease, Cleveland Clinic Florida, Weston Campus, FL, USA; 3 Division of Cardiovascular Medicine, SUNY Downstate Medical Center, Brooklyn, NY, USA; 4 Robert and Suzanne Tomsich Department of Cardiac Pacing and Electrophysiology, Department of Cardiovascular Disease, Cleveland Clinic Florida, Weston Campus, FL, USA; 5 Division of Cardiovascular Medicine, Electrophysiology Section, SUNY Downstate Medical Center, Brooklyn, NY, USA

**Keywords:** Cardiac magnetic resonance, cardiac resynchronization therapy, heart failure, meta-analysis, scar burden

## Abstract

Cardiac resynchronization therapy (CRT) has revolutionized heart failure (HF) management, offering benefits in morbidity, mortality, and symptom alleviation. However, optimal response rates are not universally achieved, necessitating enhanced patient-selection strategies. Myocardial scar patterns, quantified by delayed-enhancement cardiac magnetic resonance (DE-CMR), have been implicated in CRT outcomes. We conducted a meta-analysis of observational studies assessing CRT responses by performing a systematic literature search using PubMed, Embase, Ovid MEDLINE, Scopus, the Cochrane Library, ScienceDirect, and the Web of Science. Scar burden, left ventricular ejection fraction (LVEF), left ventricular end-systolic volume (LVESV), and left ventricular end-diastolic volume (LVEDV) were evaluated. CRT response rates among ischemic and non-ischemic cardiomyopathy patients were also explored. This meta-analysis incorporated eight studies meeting the eligibility criteria. CRT responders exhibited a significantly lower scar burden (−11.7%; 95% confidence interval, 6.6%–16.8%) compared to non-responders, supporting the predictive value of scar quantification (*I*^2^ = 95.25%; *P* < .001). Responders demonstrated an increased mean LVEF (from 25.2% to 31.9%), while non-responders showed modest changes (from 23.3% to 24.4%). Responders experienced a decrease in mean LVESV from 158.8 to 132.8 mL, contrasting with a more stable mean LVESV value in non-responders (reduction from 160.9 to 157.6 mL). Responders experienced a reduced mean LVEDV from 219.4 to 196.7 mL, while non-responders showed more minimal changes (from 213.4 to 210.6 mL). Limited data suggested a CRT response rate of 34.7% in ischemic cardiomyopathy; non-ischemic data were insufficient. In conclusion, DE-CMR, assessing the scar burden, emerges as a valuable tool for predicting the CRT response. A lower scar burden correlates with improved responses, supporting the role of DE-CMR in refining patient selection for CRT. This meta-analysis contributes insights into personalized CRT strategies, emphasizing the potential of imaging modalities to enhance therapeutic outcomes in HF patients. Further research is warranted to solidify these findings and refine clinical applications.

## Introduction

Cardiac resynchronization therapy (CRT) has emerged as a significant therapeutic intervention for heart failure (HF) patients, particularly those presenting with an intraventricular conduction delay.^1^ The benefits of CRT extend to the reduction of morbidity and mortality, the alleviation of HF symptoms, and the enhancement of exercise performance.^2^ However, despite these advancements, not all patients respond optimally to this therapy. Around 30% of patients do not derive the intended benefit, underscoring the need for improved patient-selection strategies.^2^

HF patients often display diverse patterns of myocardial scarring, even when their level of dysfunction appears similar. This heterogeneity in scar distribution and volume, or the total scar burden, may significantly affect the myocardium’s response to CRT.^3^ Prior studies have suggested that the size and location of the infarcted myocardium could be pivotal in determining the CRT response.

In light of these findings, our study sought to evaluate the utility of delayed enhancement cardiac magnetic resonance (DE-CMR) in predicting the clinical response to CRT.^4^ We aimed to ascertain the prognostic value of scar quantification by cardiac magnetic resonance (CMR) imaging in forecasting the improvement in left ventricular (LV) function post-CRT. Our study reinforces the potential of DE-MRI as a robust tool for better patient selection, which could ultimately enhance the overall CRT response rate.

## Methods

**Figure 1:**
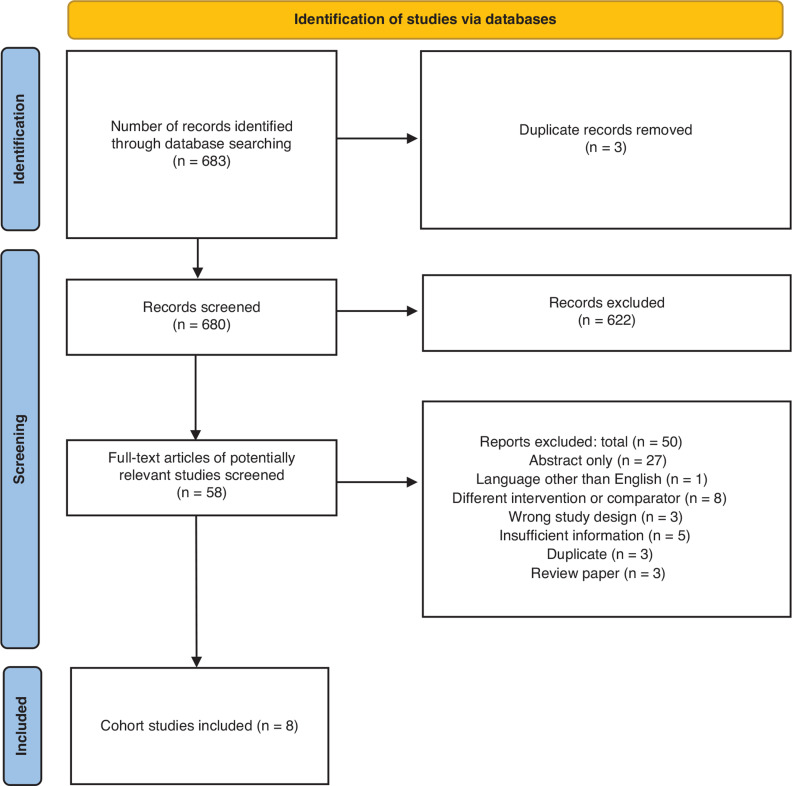
Flow diagram according to the Preferred Reporting Items for Systematic Reviews and Meta-Analyses statement, showing the identification of studies via a rigorous search of the database.

We established relevant keywords to conduct a broad search for articles related to CRT and DE-CMR in PubMed, Embase, Ovid MEDLINE, Scopus, Cochrane Science Direct, and the Web of Science published from January 1, 1990, to December 30, 2022. The literature search was inclusive and did not impose any restrictions on the publication date or language. Search combinations for each database were tailored to the database’s specific search capabilities. In total, 683 articles were retrieved. Eligible studies included observational studies, both retrospective and prospective, that evaluated clinical and/or imaging responses to CRT. We screened articles for eligibility based on their relevance. We initially screened abstracts, proceeding to a full-text review if a study’s eligibility was unclear. We finally selected studies that involved patients who received CRT as per established guidelines, underwent CMR pre- and post-CRT, and had their response to CRT assessed either clinically or through echocardiographic parameters using the LV ejection fraction (EF) (LVEF) and/or LV reverse remodeling (LVRR) by assessing the LV end-systolic volume (LVESV) and LV end-diastolic volume (LVEDV). Studies available only in abstract form were excluded. The reasons for the exclusion of other studies are detailed in the Preferred Reporting Items for Systematic Reviews and Meta-Analyses flowchart **(Figure 1)**.

Two investigators worked independently using a standardized approach to complete data extraction. In case of disagreements, a consensus was reached through discussion or with the involvement of a third independent investigator. The extracted data encompassed general information, such as lead author, publication year, and population origin, as well as specific attributes of each study, including design, sample size, participant age and sex, and prescribed medications. Additionally, we collected data on mean follow-up duration, HF etiology, and the extent of scar burden. We also recorded assessments of clinical outcomes and cardiac functions, including LVEF, LVESV, LVEDV, QRS duration, New York Heart Association (NYHA) functional class, quality of life (QoL) score, and 6-min walking distance (6MWD). Scar-related characteristics, including burden, transmurality, location, and the position of the CRT lead, were also meticulously recorded. The CRT response was based on clinical or imaging-derived parameters.

### Statistical analysis

A meta-analysis was performed to consolidate and analyze the data from the selected studies. This approach allows for the combination of findings from multiple studies to generate a single estimate of the major effects, thereby enhancing the statistical power.

The heterogeneity across the included studies was assessed using the *I*^2^ statistic. The *I*^2^ value represents the proportion of total variation across studies that is attributable to heterogeneity rather than chance. *I*^2^ > 50% is considered to indicate substantial heterogeneity. Random-effects models were used for all meta-analyses to account for the expected heterogeneity among the studies. The mean difference was used as the summary statistic for continuous outcomes, and the odds ratio (OR) was used for binary outcomes. Both of these were reported with their respective 95% confidence intervals (CIs).

Subgroup analyses were planned a priori based on factors that could influence the response to CRT, such as scar burden. *P* < .05 was considered statistically significant for all analyses. All statistical analyses were conducted using a suitable statistical software package for meta-analysis.

## Results

Our meta-analysis incorporated eight studies examining various parameters in patients undergoing CRT. Key patient characteristics at baseline are outlined in **Table 1**, while the demographics and clinical characteristics of CRT responders and non-responders are detailed in **Table 2**.

### Scar burden’s influence

**Figure 2:**
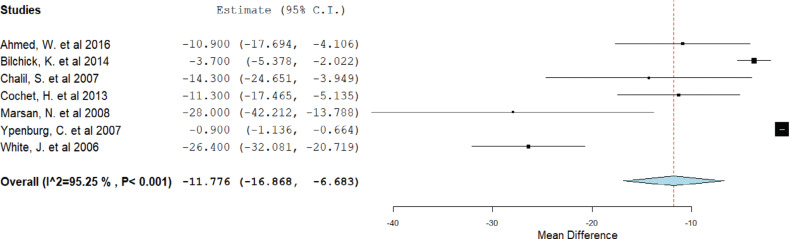
Mean scar burden difference. A forest plot showing the mean scar burden difference between cardiac resynchronization therapy responders and non-responders. *Abbreviation:* CI, confidence interval.

Incorporating data from seven studies, we identified a considerably lower scar burden among CRT responders than non-responders. The mean difference was −11.7% (95% CI, 6.6%–16.8%; heterogeneity, *I*^2^ = 95.25%; *P* < .001), revealing a significant relationship between the scar burden and the response to CRT **(Figure 2)**.

### Impact of cardiac resynchronization therapy on left ventricular ejection fraction

**Figure 3:**
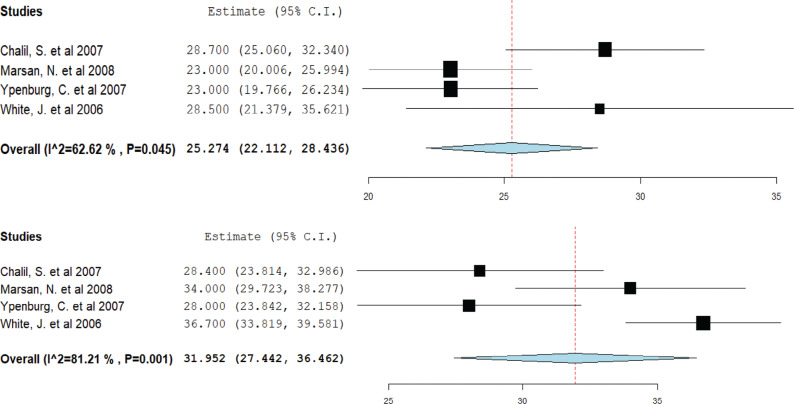
Mean ejection fraction in cardiac resynchronization therapy (CRT) responders. A forest plot showing the mean ejection fraction in CRT responders pre-CRT (upper) and post-CRT (lower). *Abbreviation:* CI, confidence interval.

**Figure 4:**
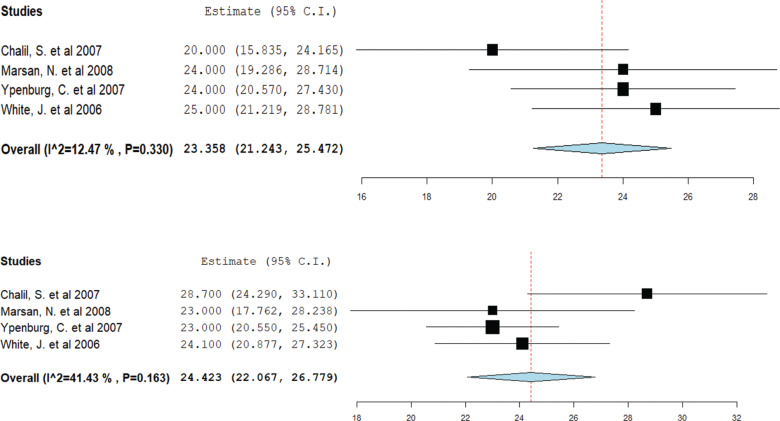
Mean left ventricular ejection fraction in cardiac resynchronization therapy (CRT) non-responders. A forest plot showing the mean ejection fraction in CRT responders pre-CRT (upper) and post-CRT (lower). *Abbreviation:* CI, confidence interval.

Four studies provided data on LV function. Responders had a mean EF of 25.2% pre-CRT, which increased to 31.9% post-CRT **(Figure 3)**. However, non-responders demonstrated a mean EF of 23.3% pre-CRT and 24.4% post-CRT **(Figure 4)**.

### Impact of cardiac resynchronization therapy on left ventricular end-systolic volume

**Figure 5:**
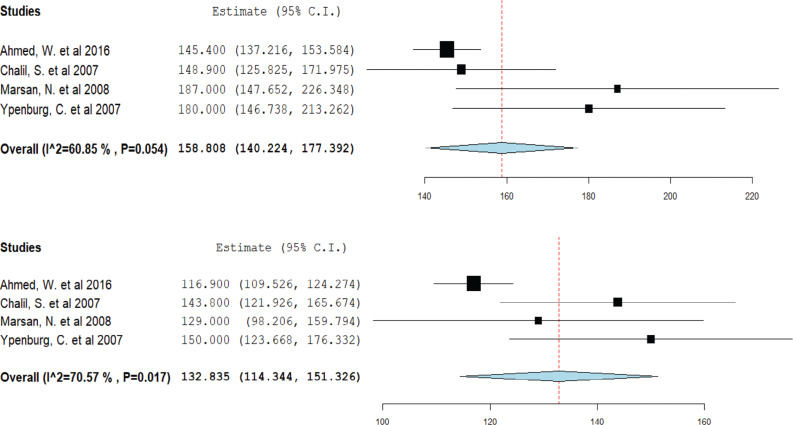
Mean left ventricular end-systolic volume in cardiac resynchronization therapy (CRT) responders. A forest plot showing the mean ejection fraction in CRT responders pre-CRT (upper) and post-CRT (lower). *Abbreviation:* CI, confidence interval.

**Figure 6:**
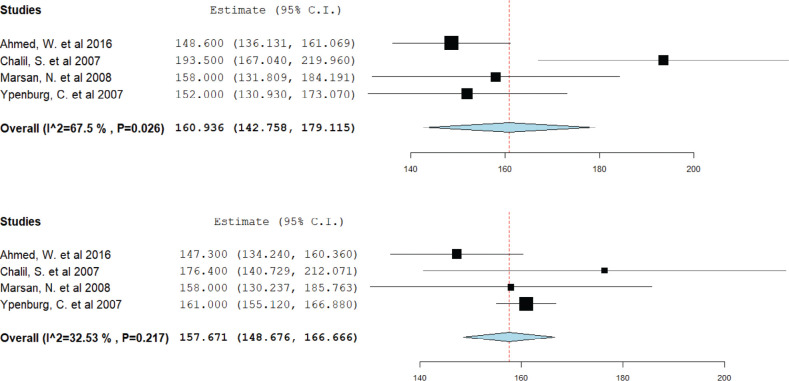
Mean left ventricular end-systolic volume in cardiac resynchronization therapy (CRT) non-responders. A forest plot showing the mean ejection fraction in CRT non-responders pre-CRT (upper) and post-CRT (lower). *Abbreviation:* CI, confidence interval.

Data from four studies were pooled for this analysis. CRT responders showed a decrease in mean LVESV from 158.8 mL pre-CRT to 132.8 mL post-CRT **(Figure 5)**. However, among non-responders, the mean LVESV remained relatively stable, decreasing only from 160.9 mL pre-CRT to 157.6 mL post-CRT **(Figure 6)**.

### Impact of cardiac resynchronization therapy on left ventricular end-diastolic volume

**Figure 7:**
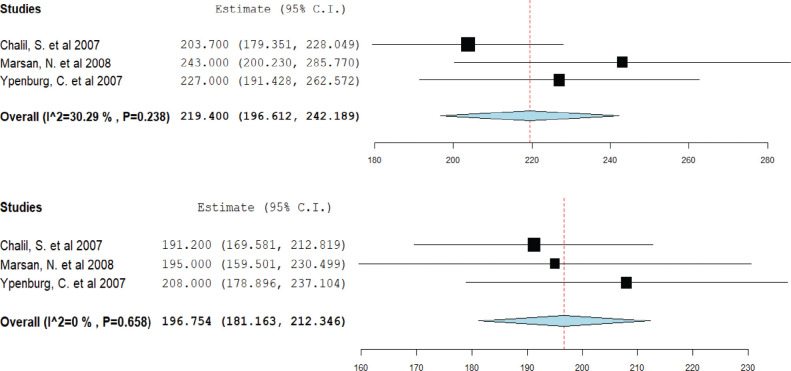
Mean left ventricular end-diastolic volume in cardiac resynchronization therapy (CRT) responders. A forest plot showing the mean ejection fraction in CRT responders pre-CRT (upper) and post-CRT (lower). *Abbreviation:* CI, confidence interval.

**Figure 8:**
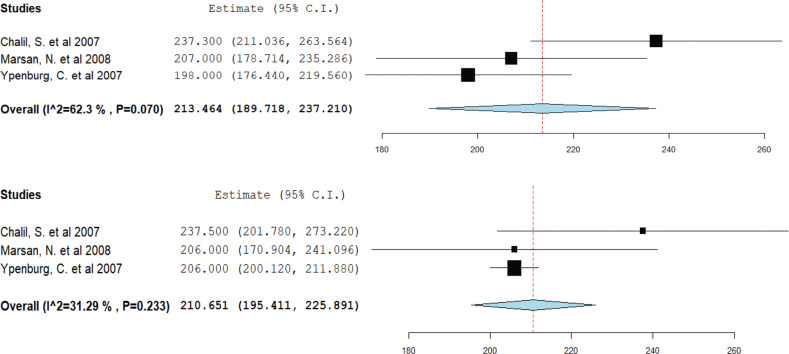
Mean left ventricular end-diastolic volume in cardiac resynchronization therapy (CRT) non-responders. A forest plot showing the mean ejection fraction in CRT non-responders pre-CRT (upper) and post-CRT (lower). *Abbreviation:* CI, confidence interval.

Three studies contributed to this analysis. Responders demonstrated a reduction in mean LVEDV from 219.4 mL pre-CRT to 196.7 mL post-CRT **(Figure 7)**. In contrast, non-responders showed a minimal change in mean LVEDV, from 213.4 mL pre-CRT to 210.6 mL post-CRT **(Figure 8)**.

### Impact of heart failure etiology

**Figure 9:**
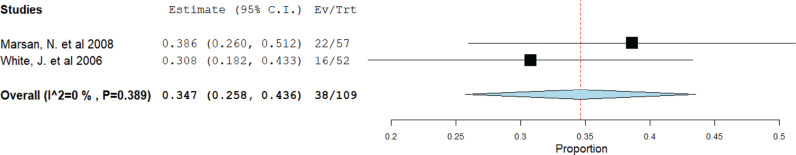
A forest plot showing the average cardiac resynchronization therapy response rate in patients with ischemic cardiomyopathy. *Abbreviations:* CI, confidence interval; Ev/Trt, event/treated.

From two studies, we derived an average CRT response rate of 34.7% in patients with ischemic cardiomyopathy **(Figure 9)**. However, we lacked sufficient data to estimate the CRT response rate among patients with non-ischemic cardiomyopathy.

### Summary of previous studies

The 2006 study by White et al.^5^ examined the ability of DE-MRI to predict the clinical response to cardiac CRT. The study was conducted on 23 HF patients undergoing CRT. The patients had LVEFs of ≤35%, NYHA functional class II–IV disease, and interventricular dyssynchrony of ≥60 ms. The scar measured by planimetry was correlated with the response criteria. Responders were those who showed a positive change in the following parameters: LVEF, QoL score, 6MWD, and NYHA functional class. On the contrary, non-responders were those who did not show significant improvements in these parameters. The results showed that the percentage of the total scar was significantly higher in the non-response group (10 patients) compared to the response group (13 patients), with a median (interquartile range) of 24.7% (18.1%–48.7%) versus 1.0% (0.0%–8.7%) (*P* = .0022). The DE-MRI accurately predicted the clinical response to CRT, suggesting that this technique offers unique and predictive information in the assessment of patients referred for CRT. The study also found that patients with systolic HF have heterogeneous patterns of myocardial scarring despite similar alterations in contractile function. The volume, location, and transmurality of this scarring are similarly heterogeneous between individuals. The study suggests that the amount and distribution of myocardial scar may be an important determinant of the response to CRT.

The 2007 study by Ypenburg et al.^6^ evaluated the influence of scar burden on the response to CRT. The study included 34 patients with ischemic cardiomyopathy. The researchers used contrast-enhanced magnetic resonance imaging (MRI) to determine the total scar burden. They used a 17-segment model with a 5-point hyperenhancement scale to score the scar burden, with a score of 0 points indicating no scar and a score of 4 points indicating a transmural scar (hyperenhancement > 76%). Responders were defined as those who showed a decrease of ≥10% in LVESV after 6 months of CRT. The study found that 18 patients (53%) were responders and 16 (47%) were non-responders. They found a significant inverse relation between the total scar burden and reverse remodeling (RR) (*r* = −0.91; *P* < .05). The more extensive the scar burden, the lower the likelihood of LVRR after CRT. Furthermore, patients not responding to CRT had significantly more scar tissue than responders. A scar burden of >1.20 resulted in a complete functional non-response. They concluded that, the more extensive the scar burden, the lower the likelihood of LVRR after.

The 2007 study by Chalil et al.^4^ aimed to determine whether myocardial scarring, quantified using DE-CMR, predicts the response to CRT. Their study involved a total of 45 patients with ischemic cardiomyopathy. Participants underwent an assessment of 6MWD and QoL before and after CRT. The scar size (percentage of LV mass), location, and transmurality were assessed prior to CRT using DE-CMR. The response to CRT was defined as survival for 1 year with no HF hospitalizations and improvement by ≥1 NYHA class or ≥25% in 6MWD. Responders had a higher LVEF, smaller scars (<33%), and fewer scars with ≥51% transmurality. The scar size correlated negatively with changes in 6MWD and positively with changes in QoL scores. The responder rates in patients with <33% scar were greater than in those with ≥33% scar (82% vs. 35%). The responder rates in patients with scar transmurality of <51% were higher than in those with scar transmurality of ≥51% (89% vs. 46%). Among the patients with posterolateral scars, a transmurality value of ≥51% was associated with a particularly poor response rate (23%) when compared to scars with <51% transmurality (88%). In the multivariate analyses, both the scar size and transmurality emerged as predictors of response. In patients with posterolateral scars, pacing outside the scar was associated with a better responder rate than pacing over the scar (86% vs. 33%). The exact numbers of patients in the response and non-response groups are not explicitly stated in the extracted information. However, based on the response rates provided, it can be inferred that most patients responded to CRT, especially those with smaller scar sizes and less transmurality.

The 2009 study by Marsan et al.^7^ assessed the relative value of a novel measure of LV dyssynchrony derived from MRI and the extent of scar tissue for the prediction of response to CRT. The study included 35 HF patients scheduled for CRT. The response to CRT was defined based on a reduction of ≥15% in LVESV 6 months after implantation. At the 6-month follow-up, 21 patients (60%) were classified as responders, while the remaining 14 patients (40%) were classified as non-responders. The investigators found that, on MRI, the standard deviation of 16-segment time-to-maximum radial wall thickness, a measure of LV dyssynchrony, was significantly greater in responders compared with non-responders (median, 97 vs. 60 ms; *P* < .001). This suggests that a greater degree of LV dyssynchrony was associated with a better response to CRT. On the contrary, the total extent of scar tissue was larger in non-responders (median, 35% in non-responders vs. 3% in responders; *P* < .001). Their conclusion suggests that both LV dyssynchrony and the extent of scar tissue are important predictors of echocardiographic response to CRT.

The 2013 study by Cochet et al.^8^ aimed to identify the predictors of RR after CRT using MRI. The study involved 60 patients. The response group consisted of 43 patients who showed a reduction in LVESV of >15% at 6 months post-CRT, while the non-response group included 17 patients who did not show this reduction. The study found that the presence of myocardial scar and intra-LV dyssynchrony were significant predictors of RR after CRT. Patients with a greater extent of myocardial scar were less likely to respond to CRT. Similarly, patients with significant intra-LV mechanical dyssynchrony were more likely to respond to CRT. The response to CRT was defined based on the change in LVESV. A reduction of >15% in ESV at 6 months post-CRT was considered a response, while a reduction of <15% was considered a non-response. The study also found a discrepancy between electrical and mechanical dyssynchrony. All patients showed significant electrical dyssynchrony as measured by QRS duration on the surface electrocardiogram. However, 17 out of 60 patients did not show significant intra-LV mechanical dyssynchrony. This study suggests that patients with electrical but not mechanical dyssynchrony are less likely to respond to CRT. The presence of scar at the pacing site, even when non-transmural, was associated with less RR after CRT, whereas mechanical delay at this site did not seem to influence the response. The authors concluded that MRI-defined intra-LV dyssynchrony and myocardial scar extent are independent predictors of RR at 6 months after CRT. The presence of scar at the pacing site is associated with a reduced response to CRT, whereas mechanical delay at this site does not seem to influence the response.

The 2014 study by Bilchick et al.^9^ aimed to evaluate the relative influences of mechanical, electrical, and scar properties at the LV lead position (LVLP) on CRT response and clinical events using CMR. The study involved a cohort of 75 patients who had either a class I or class IIa indication for CRT according to the current guidelines and underwent implantation of a CRT defibrillator with subsequent clinical follow-up. The median follow-up period was 2.6 years. CRT response was defined as a 15% reduction in LVESV. Based on this definition, the study identified 40 patients (53%) as CRT responders. They found that mechanical, electrical, and scar properties at the LVLP together with CMR mechanical dyssynchrony are strongly associated with echocardiographic CRT response and clinical events after CRT. In their multivariable logistic modeling, they found that the odds of a CRT response were influenced by several factors: circumferential uniformity ratio estimate (CURE) (OR, 2.59/0.1 decrease), delayed circumferential contraction onset at LVLP (OR, 6.55), absence of LVLP scar (OR, 14.9), and time from QRS onset to LVLP electrogram (OR, 1.31/10-ms increase). The study authors also determined that patients with CUREs of <0.70, absence of LVLP scar, and delayed LVLP contraction onset had a 100% response rate, whereas those with CUREs of ≥0.70 had a 0% CRT response rate and a 12-fold increased risk of death. In terms of clinical outcomes, during the follow-up period, 21.3% of patients died, 16.0% had sustained ventricular tachycardia or fibrillation, and 26.7% were hospitalized with HF. The rates of all these events were much higher among non-responders compared to responders.

The 2016 study by Ahmed et al.^10^ aimed to investigate the impact of LV scar on the outcomes of CRT. The study enrolled 30 patients who underwent CRT implantation. The assessment of LV dyssynchrony was done through gated single-photon emission computed tomography (SPECT) LV phase analysis. Before implantation, DE-CMR was used to examine LV scar burden. An echocardiographic examination of LVESV was performed prior to CRT and 6 months later. Out of the 30 patients who received CRT (mean age, 58.7 ± 9.0 years; 24 men), reverse LV remodeling (decline of ≥15% from baseline LVESV) was documented in 19 patients, who were classified as responders. The remaining 11 patients did not show this level of decline and were classified as non-responders. The authors found that temporal changes in LV dyssynchrony parameters correlated with LVRR. Completing receiver operating characteristic curve analysis for predicting a CRT non-response revealed a cutoff of 36.5% of global LV scar burden, which had a sensitivity of 81.8% and a specificity of 68.4%. A cutoff for lateral wall scar burden of 40.5% of the whole lateral wall had a sensitivity of 72.7% and a specificity of 68.4%. The authors concluded that reverse LV remodeling is associated with temporal improvements in LV dyssynchrony parameters, that LV scar had an unfavorable impact on CRT response, and that both global and lateral wall scar burden can predict CRT non-response status.

This 2019 study by Harb et al.^11^ investigated the impact of myocardial scar burden on the response to CRT. The study defined responders and non-responders based on the clinical and echocardiographic outcomes post-CRT. Clinical response was defined based on the occurrence of adverse events post-CRT. Adverse events included HF admission or death. A total of 29 patients experienced an adverse event during follow-up (HF in 19 and death in 10). Echocardiographic response was defined as an increase by ≥10% in LVEF, ≥3 months after CRT implantation. Overall, 30 patients (53%) showed a significant EF improvement (ie, an increase in EF by ≥10% after CRT), while 27 (42%) did not. The authors found that the presence of a scar in any location (anterior, inferior, lateral, or septal) was associated with worse outcomes, both in terms of LV function improvement and clinical events. Furthermore, a subset of patients may experience worsening LV function after CRT. The study also found that total scar percentage was significantly higher in patients who had an event compared with those who were event-free for the primary endpoint (22% vs. 12%; *P* = .02). After adjusting for the presence of left bundle branch block and QRS duration as well as clinical and LV lead characteristics, older age and greater degrees of scar were associated with a higher risk of the composite clinical endpoint (HR per 1% increase in scar, 1.06; 95% CI, 1.02–1.10; *P* < .001). The authors concluded that scar burden is an independent and incremental predictor of CRT response, and it can be used to improve patient selection for CRT.

## Discussion

Our meta-analysis synthesized data from eight studies to evaluate the impact of CRT on various parameters in patients. A significant finding was the lower scar burden among CRT responders compared to non-responders, with a mean difference of −11.7%, suggesting a strong correlation between scar burden and CRT response. In terms of LV function, CRT responders exhibited a notable improvement in LVEF, from a mean of 25.2% pre-CRT to 31.9% post-CRT. Conversely, non-responders showed a minimal change, with a mean LVEF of 23.3% pre-CRT and 24.4% post-CRT. A similar pattern was observed in LVESV and LVEDV. CRT responders experienced a decrease in mean LVESV from 158.8 mL pre-CRT to 132.8 mL post-CRT and a reduction in mean LVEDV from 219.4 mL pre-CRT to 196.7 mL post-CRT. Non-responders, however, showed little change in these parameters, with LVESV decreasing from 160.9 mL pre-CRT to 157.6 mL post-CRT and LVEDV decreasing from 213.4 mL pre-CRT to 210.6 mL post-CRT. Finally, our analysis revealed a CRT response rate of 34.7% in patients with ischemic cardiomyopathy. However, due to limited data, we were unable to estimate the CRT response rate in patients with non-ischemic cardiomyopathy.

Our study has taken a step forward in the field of CRT by demonstrating the significance of scar burden ascertained by DE-MRI in predicting the CRT response. Our study confirmed that patients with a lower scar burden were more likely to respond positively to CRT. This finding aligns with a growing body of evidence suggesting that a comprehensive evaluation of myocardial scarring can facilitate a more nuanced approach to predicting the CRT response.^12^

Our findings are in line with those of prior studies, including the aforementioned ones by Chalil et al., White et al., Ypenburg et al., Marsan et al., Cochet et al., Bilchick et al., and Ahmed et al.,^4–10^ all of which also highlighted the significant impact of myocardial scarring on CRT response. These studies collectively emphasize that the response to CRT is not a uniform phenomenon but varies depending on individual patient characteristics—specifically, the degree and location of myocardial scarring.^13,14^

Interestingly, prior studies using echocardiography, SPECT, or MRI conclude that intra-LV mechanical dyssynchrony and myocardial scar extent are independent predictors of RR after CRT.^15^ We found that myocardial scar extent predicts CRT response independently from other characteristics, including mechanical dyssynchrony. This suggests that the assessment of myocardial scar extent might be well suited to the identification of potential responders beyond the current indication criteria, and that scar assessment by CMR has promise for refining the criteria for the identification of probable non-responders, and CMR-guided CRT placement away from the scar location may also result in better clinical outcomes.^16^

By considering myocardial scar burden in addition to traditional indicators, clinicians can potentially identify patients who are likely to benefit from CRT and avoid unnecessary and costly interventions in those who are less likely to respond.

### Limitations

While our systematic review and meta-analysis provide important insights into the use of MRI for predicting the response to CRT, several limitations exist. As a retrospective study, our findings are subject to potential biases, including sampling error, selection, and publication bias. The studies included in our analysis used different methods and criteria to assess the CRT response, which could influence the results. Our reliance on the accuracy and completeness of the original studies’ data could also impact our findings. Additionally, we did not have access to individual patient data, limiting our ability to perform detailed analyses or to control for potential confounding factors. Our conclusions about the role of myocardial scar extent in identifying potential CRT responders are based on retrospective data and need confirmation through prospective clinical trials.

## Conclusion

Our study underscores the pivotal role of myocardial scar burden, as assessed by DE-MRI, in predicting the response to CRT. Our findings align with and expand upon the existing literature, demonstrating that patients with a lower scar burden tend to exhibit a positive response to CRT, while those with a larger extent of scar tissue respond less favorably. The inclusion of scar burden assessment in the process of patient selection for CRT can potentially enhance the predictive accuracy of CRT response, thereby optimizing the benefits of this therapy. Future research should continue to refine our understanding of the complex interplay between myocardial scarring, LV dyssynchrony, and CRT response.

## Figures and Tables

**Table 1:** Baseline Study Characteristics of All Included Studies

Authors	Country	Year	Design	Inclusion Criteria	Exclusion Criteria	Sample Size (n)	
						Responders	Non-responders
Chalil et al.^4^	UK	2007	Single-center, uncontrolled, observational study	(1) HF because of coronary heart disease; (2) NYHA class III or IV; (3) attendance to a dedicated HF clinic to achieve maximum tolerated treatment with diuretics, ACE-I, ARB, β-blocker, spironolactone, and digoxin; (4) LBBB, with a QRS duration of ≥120 ms; and (5) LVEF ≤ 35%	(1) Contraindications to cardiac pacing, (2) MI or ACS within the previous 3 months, (3) presence of or indications for an ICD, (4) severe structural valvular heart disease, (5) presence of comorbidities likely to threaten survival for 12 months, (6) pulmonary edema requiring IV diuretics in the previous week, and (7) intermittent or chronic atrial fibrillation or atrial flutter	29	16
White et al.^5^	Canada	2006	Prospective study	(1) Symptomatic congestive HF NYHA functional class II or higher, (2) LVEF ≤ 35% as measured by two-dimensional echocardiography or radionuclide angiography, (3) QRS duration of ≥120 ms, and (4) intraventricular dyssynchrony ≥60 ms	MI within 1 month, revascularization procedure within 3 months, and standard contraindications to MRI imaging	13	10
Ypenburg et al.^6^	The Netherlands	2007	Prospective study	NYHA class III or IV, LVEF < 35%, and QRS duration > 120 ms	Recent MI (<3 months) or presentation with decompensated HF; patients with pacemakers or intracranial clips	53	47
Marsan et al.^7^	The Netherlands	2009	Prospective study	NYHA functional class III–IV despite optimal medical therapy, LVEF ≤ 35%, and QRS duration ≥ 120 ms	Recent MI (<3 months), decompensated HF, previous cardiac pacemaker/ICD or intracranial clips, atrial fibrillation, claustrophobia	21	14
Cochet et al.^8^	France	2013	Prospective study	(1) LVEF ≤ 35% at echocardiography, NYHA functional class III or IV, and QRS duration ≥ 120 ms or (2) NYHA functional class II and QRS duration ≥ 150 ms	Contraindications to gadolinium-enhanced MRI, presence of permanent atrial fibrillation, or history of atrial fibrillation	42	18
Bilchick et al.^9^	USA	2014	Prospective study	Clinical indication for CRT based on established guidelines and a GFR of ≥45 mL/min/1.73 m^2^ in order to receive gadolinium	Not specified	40	35
Ahmed et al.^10^	Egypt	2016	Prospective single-center study	Eligible for CRT implantation according to ACCF/AHA guidelines for managing HF	Recent MI of <3 months or dysrhythmias that could result in gating artifacts	19	11
Harb et al.^11^	USA	2019	Retrospective single-center study	All consecutive patients who underwent CMR testing between January 2002 and June 2014 and had subsequent CRT implantation were initially included	Patients who underwent CRT-P implant without CRT-D implant and those with significant time delay between the CMR scan and CRT implantation (>1 year for ICM and >2 years for NICM) were excluded	30	27

*Abbreviations:* ACE-I, angiotensin-converting enzyme inhibitor; ACS, acute coronary syndrome; ARB, angiotensin-II receptor blocker; CMR, cardiac magnetic resonance; CRT, cardiac resynchronization therapy; CRT-D, cardiac resynchronization therapy defibrillator; CRT-P, cardiac resynchronization therapy pacemaker; GFR, glomerular filtration rate; HF, heart failure; ICD, implantable cardioverter-defibrillator; IV, intravenous; LBBB, left bundle branch block; LVEF, left ventricular ejection fraction; MI, myocardial infarction; MRI, magnetic resonance imaging; NYHA, New York Heart Association.

**Table 2:** Demographic and Clinical Characteristics of Cardiac Resynchronization Therapy Responders and Non-responders

Authors	Age (Mean ± SD) (Years)			Sex (%)				Follow-up Duration (Months)	Definition of Response
	All	Responders	Non-responders	Responders		Non-responders			
				Male	Female	Male	Female		
Chalil et al.^4^	67.1 ± 10.4	65.5 ± 10.6	69.9 ± 9.7	83	17	88	12	17.7	(1) Survival for 1 year following implantation, (2) no hospitalizations for HF for 1 year following implantation, and (3) improvement by ≥1 NYHA class or by ≥25% in 6MWD result
White et al.^5^	64.9 ± 11.7	66.2 ± 10.4	63.2 ± 13.6	62	38	70	30	3	(1) Increase in LVEF by ≥5%, (2) improvement in NYHA functional class by ≥1 class, (3) improvement in 6MWD result by ≥30 m, and (4) decrease in Minnesota Living With Heart Failure score by ≥10 points. Clinical response was defined as an improvement in either EF or 6MWD result plus ≥1 other response criteria at follow-up.
Ypenburg et al.^6^	68 ± 10	Not specified	Not specified	Not specified	Not specified	Not specified	Not specified	6	A significant improvement in clinical parameters after CRT; improvement in LVEF with RR
Marsan et al.^7^	Not specified	64 ± 9	62 ± 12	76	23	71	28	6.5	Reduction of ≥15% in LVESV 6 months after implantation on TTE
Cochet et al.^8^	59 ± 11	60 ± 10	57 ± 11	74	26	72	28	6	Positive response was defined as RR ≥ 15% of baseline LVESV. Super-response was defined as LVEF normalization at 6 months, ie, >50%, as measured with echocardiography. A positive clinical response at 6 months was defined by an NYHA class improvement (≥1 class) without hospitalization for HF during follow-up.
Bilchick et al.^9^	65.9	65.3	67.9	60	40	91	9	31.2	15% reduction in LVESV at 6 months (or the last follow-up echocardiogram prior to death if the patient died prior to 6 months after implantation)
Ahmed et al.^10^	58.7 ± 9.0	55.3 ± 10.6	57.7 ± 9.8	74	26	90	10	6	≥15% decrease in LVESV from initial baseline measurements, ie, reverse LV remodeling
Harb et al.^11^	62 ± 12	Not specified	Not specified	Not specified	Not specified	Not specified	Not specified	44.4	An increase by ≥10% in EF ≥3 months after CRT implantation

*Abbreviations:* 6MWD, 6-min walking distance; CRT, cardiac resynchronization therapy; HF, heart failure; LVEF, left ventricular ejection fraction; LVESV, left ventricular end-systolic volume; NYHA, New York Heart Association; RR, reverse remodeling; TTE, transthoracic echocardiography.
